# Characterization and fungicides sensitivity of *Colletotrichum* species causing *Hydrangea macrophylla* anthracnose in Beijing, China

**DOI:** 10.3389/fpls.2024.1504135

**Published:** 2025-01-21

**Authors:** Juan Zhao, Yanli Cheng, Yayong Liu, Xiaojing Shi, Taotao Zhang, Wentao Qin

**Affiliations:** ^1^ Institute of Plant Protection, Beijing Academy of Agriculture and Forestry Sciences, Beijing, China; ^2^ Beijing Key Laboratory of Environment Friendly Management on Fruit Diseases and Pests in North China, Beijing Academy of Agriculture and Forestry Sciences, Beijing, China; ^3^ College of Life Sciences, Yangtze University, Jingzhou, Hubei, China; ^4^ Department of Biology, Xinzhou Normal University, Xinzhou, Shanxi, China

**Keywords:** *Hydrangea macrophylla*, anthracnose, *Colletotrichum* species, multi-loci phylogeny, fungicide sensitivity

## Abstract

*Hydrangea macrophylla* (Thunb.) Ser. is one of the widely cultivated plants in home gardens and scenic areas of China. Anthracnose disease is commonly observed during the normal growth of *H. macrophylla*, significantly impacting its ornamental and economic values. From 2021 to 2023, an investigation on *H. macrophylla* anthracnose was carried out in nine parks of Beijing, China, and a total of 114 *Colletotrichum* isolates were obtained from the diseased leaves with typical anthracnose symptoms. Based on morphological characteristics and phylogenetic analysis of six genomic loci including rDNA-ITS, *ACT*, *TUB2*, *CAL*, *CHS-1*, and *GAPDH*, these isolates were identified as belonging to six *Colletotrichum* species. Among which, *C. gloeosporioides* was the most abundant (65 isolates, 57.0%), followed by *C. fructicola* (33 isolates, 28.9%), *C. aenigma* (8 isolates, 7.0%), *C. truncatum* (4 isolates, 3.5%), *C. subacidae* (2 isolates, 1.8%) and *C. sojae* (2 isolates, 1.8%). Pathogenicity test conducted on detached leaves of *H. macrophylla* revealed a distinct variation in virulence among isolates from different *Colletotrichum* species, and wounding was either essential or conducive to successful infection. Specifically, *C. gloeosporioides* exhibited greater aggressiveness, resulting in larger lesions, while *C. subacidae* induced lesions most quickly. Fungicide sensitivity assays demonstrated that prochloraz exerted a remarkable inhibitory effect on the mycelial growth of representative isolates belonging to the three predominant *Colletotrichum* species. In contrast to difenoconazole and tebuconazole, the mean EC_50_ values for prochloraz against *C. gloeosporioides*, *C. fructicola*, and *C. aenigma* were 0.062, 0.033, and 0.023 μg/ml, respectively. This is the first report of *C. aenigma*, *C. truncatum*, *C. subacidae* and *C. sojae* causing *H. macrophylla* anthracnose worldwide including China. These findings have elucidated the *Colletotrichum* species associated with *H. macrophylla* anthracnose as well as their fungicides sensitivities in Beijing, China. This provides a scientific foundation for the accurate diagnosis and local management of *H. macrophylla* anthracnose.

## 1 Introduction

*Hydrangea macrophylla* (Thunb.) Ser., commonly known as an ornamental garden plant of the *Hydrangea* genus, enjoys great popularity due to its sizable inflorescence and abundant variety of flower colors (Mmbaga et al., 2012). The economic values have stimulated an increasing demand for cultivation and management of *H. macrophylla*, with particular emphasis on disease diagnosis and management (Wu et al., 2021). As one of the typical woody plants worldwide, *H. macrophylla* is susceptible to many diseases caused by a variety of fungal pathogens throughout the growth period, such as *Alternaria alternata* (Garibaldi et al., 2007; Liu et al., 2017), *Cladosporium tenuissimum* (Li et al., 2021), *Colletotrichum* spp. (Mmbaga et al., 2012), *Corynespora cassiicola* (Mmbaga et al., 2012), and *Phoma exigua* (Garibaldi et al., 2006). Among which, anthracnose is one of the most important fungal diseases on *H. macrophylla*. It usually affects the leaves and flowers, leading to seriously influence on the ecological landscape construction.

Plant anthracnose is commonly caused by species within the genus *Colletotrichum*, which is a large group of plant pathogenic fungi and is ranked eighth among the top ten fungal plant pathogens worldwide (Dean et al., 2012). Morphological characteristics comparison and multi-loci sequence analyses have been highly valued by mycologists for identifying *Colletotrichum* species. To date, a total number of about 280 species have been clarified (Dowling et al., 2020; Liu et al., 2022). However, the composition of *Colletotrichum* populations and the dominant pathogenic species responsible for anthracnose varied among different plants (Guarnaccia et al., 2021; Zhou et al., 2023). Currently, four *Colletotrichum* species, namely *C. gloeosporioides* (Mmbaga et al., 2012), *C. fructicola* (Guarnaccia et al., 2021), *C. dematium* (Maheswari et al., 2015), *C. siamense* (Hu et al., 2023) have been documented on *H. macrophylla* anthracnose worldwide. Among which, *C. gloeosporioides* has been identified as a pathogen causing *H. macrophylla* anthracnose in Qinhuangdao, Hebei province, China (Qi et al., 2008) and in Kaili, Guizhou province, China (Zhang et al., 2016). Meanwhile, *C. siamense* was reported as the causal agent of *H. macrophylla* anthracnose in Nanchang, Jiangxi province, China (Hu et al., 2023). A more comprehensive understanding of the dominant *Colletotrichum* species associated with *H. macrophylla* anthracnose will provide a foundation for developing novel strategies to manage the disease. However, the specific *Colletotrichum* taxa responsible for *H. macrophylla* anthracnose remained unknown in Beijing, China.

QoIs and DMIs are currently the major groups of fungicides employed for controlling anthracnose in agricultural crops (Kongtragoul et al., 2020; Wang et al., 2020). Certain *Colletotrichum* species have exhibited varying degrees of reduced sensitivity or even resistance to such fungicides (Shi et al., 2020). However, in actual production of *H. macrophylla*, unified management measures were frequently implemented but without significant efficacy. Moreover, the sensitivity of *Colletotrichum* species associated with *H. macrophylla* anthracnose to the commonly used fungicides were still uncertain. Thus, the aim of this study was thus (1) to identify the diversity of *Colletotrichum* species associated with *H. macrophylla* anthracnose by analyzing morphological characteristics coupled with multi-loci phylogenetic analysis, (2) to validate the pathogenicity and virulence of the identified *Colletotrichum* species by fulfilling Koch’s postulates, (3) to evaluate the sensitivity of the dominant *Colletotrichum* species to the commonly used fungicides, thereby providing a theoretical foundation for disease diagnosis and scientific management of anthracnose on *H. macrophylla* in Beijing, China.

## 2 Materials and methods

### 2.1 Field investigation and sample collection

From the summer of 2021 to 2023, investigations into the incidence and severity of anthracnose were carried out in *H. macrophylla* growing nurseries of nine parks in Beijing, China (**Table 1**). Disease incidence was calculated as the proportion of *H. macrophylla* plants exhibiting anthracnose symptoms relative to the total number of plants under evaluation. A total of 46 leaf samples of *H. macrophylla* exhibiting typical symptoms of anthracnose were collected for further study. Small pieces (5*5 mm) of leaf tissues were cut from the margin of anthracnose lesions, surface sterilized with 70% ethanol for 30 s, 1% NaClO for 30 s, then rinsed in sterile distilled water for three times, finally transferred onto potato dextrose agar (PDA) with lactic acid (0.1%) and incubated at 28°C for 3 days. Subsequently, the growing edges of the fungal colonies were aseptically transferred onto new PDA plates. The obtained fungal isolates were purified by single spore method and then reserved in 25% (v/v) glycerol at -80°C. The prevalence of *Colletotrichum* species was estimated as isolation rate (RI) and calculated using the formula RI% = (NS/NI) × 100%, Where NS is the number of isolates belonging to a specific species and NI is the total number of isolates (Vieira et al., 2014). All the 114 *Colletotrichum* isolates were deposited in the Laboratory of Biocontrol Microorganisms, Institute of Plant Protection, Beijing Academy of Agricultural and Forestry Sciences.

**Table 1:** Sample sites of *Hydrangea macrophylla* leaves with anthracnose symptoms and prevalence of the obtained *Colletotrichum* species.

Sample sites		Xiangshan Park	Xishan Park	Daoxianghu Park	Baiwang Park	Shuguang Park	Banjing Road	Wufu Park	Mentougou Park	Fengtai Park	In total
Sample numbers		3	8	4	3	6	5	5	6	6	46
*C. gloeosporioides* species complex	*C. gloeosporioides*	4	11	4	7	11	8	7	6	7	65
	*C. fructicola*	2	5	2	4	6	0	5	4	5	33
	*C. aenigma*	1	1	1	1	0	1	0	2	1	8
*C. truncatum* species complex	*C. truncatum*	0	0	0	1	1	0	1	1	0	4
	*C. subacidae*	0	1	0	1	0	0	0	0	0	2
*C. orchidearum* species complex	*C. sojae*	0	0	0	0	1	1	0	0	0	2
In total		7	18	7	14	19	10	13	13	13	114

### 2.2 DNA extraction, PCR amplification and phylogenetic analysis

Genomic DNA of the fungal isolates was extracted using the Solarbio^®^ Fungi Genomic DNA Extraction Kit (Solarbio, China) according to the manufacturer’s instructions. The internal transcribed spacer region of ribosomal DNA (rDNA-ITS) was amplified using PCR primers ITS1 and ITS4 in order to screen the *Colletotrichum* sp. (White et al., 1990). For further precise identification, the representative *Colletotrichum* sp. were subjected to amplification of the partial sequences of actin (*ACT*) (Carbone and Kohn, 1999), beta tubulin (*TUB2*) (Glass and Donaldson, 1995; O’Donnell and Cigelnik, 1997), Calmodulin (*CAL*) (Weir et al., 2012), chitin synthase (*CHS-1*) (Carbone and Kohn, 1999), glyceraldehyde-3-phosphate dehydrogenase (*GAPDH*) (Templeton et al., 1992) genes with the corresponding primers listed in **Supplementary Table S1**. The amplifications were performed in a 25 μL mixture containing 10.5 μL ddH_2_O, 12.5 μL 2×PCR MasterMix, 1 μL DNA template, and 0.5 μL of each primer (10 μM) as described by Weir et al. (2012). DNA sequencing was performed by Beijing B&M Biotech Co., Ltd., China, using forward and reverse primers. Sequences were subjected to BLAST searches and submitted in the National Center for Biotechnology Information (NCBI) database with accession numbers of PP709475-PP709509 for rDNA-ITS, PP768443-768477 for *ACT*, PP768478-768512 for *TUB2*, PP768513-768547 for *CAL*, PP768548-768582 for *CHS-1*, PP768583-PP768617 for *GAPDH*, respectively (**Table 2**).

**Table 2:** Information of 35 representative *Colletotrichum* isolates from *Hydrangea macrophylla* anthracnose used for morphological characterization, phylogenetic analysis and pathogenicity test.

Species	Isolates numbers	Origins	GenBank accession number					
			rDNA-ITS	*ACT*	*TUB2*	*CAL*	*CHS-1*	*GAPDH*
*C. gloeosporioides*	JZB1040-3-1	Fengtai Park	PP709475	PP768443	PP768478	PP768513	PP768548	PP768583
	JZB1040-10-3	Xiangshan Park	PP709476	PP768444	PP768479	PP768514	PP768549	PP768584
	JZB1241-2-5	Baiwangshan Park	PP709477	PP768445	PP768480	PP768515	PP768550	PP768585
	JZB1241-3-2	Baiwanshang Park	PP709478	PP768446	PP768481	PP768516	PP768551	PP768586
	JZB1493-2-1	Xishan Park	PP709479	PP768447	PP768482	PP768517	PP768552	PP768587
	JZB1493-4-1	Xishan Park	PP709480	PP768448	PP768483	PP768518	PP768553	PP768588
	JZB1494-6-1	Xishan Park	PP709481	PP768449	PP768484	PP768519	PP768554	PP768589
	JZB1372-1-4	Wufu Park	PP709482	PP768450	PP768485	PP768520	PP768555	PP768590
	JZB1553-1-1	Banjing Road	PP709483	PP768451	PP768486	PP768521	PP768556	PP768591
	JZB1124-2-3	Shuguang Park	PP709484	PP768452	PP768487	PP768522	PP768557	PP768592
	JZB1558-2-1	Daoxianghu Park	PP709485	PP768453	PP768488	PP768523	PP768558	PP768593
	JZB1562-1-3	Qianlingshan Park	PP709486	PP768454	PP768489	PP768524	PP768559	PP768594
*C. fructicola*	JZB1040-6-4	Fengtai Park	PP709487	PP768455	PP768490	PP768525	PP768560	PP768595
	JZB1040-10-1	Xiangshan Park	PP709488	PP768456	PP768491	PP768526	PP768561	PP768596
	JZB1241-2-1	Baiwangshan Park	PP709489	PP768457	PP768492	PP768527	PP768562	PP768597
	JZB1241-2-7	Baiwangshan Park	PP709490	PP768458	PP768493	PP768528	PP768563	PP768598
	JZB1125-2-5	Shuguang Park	PP709491	PP768459	PP768494	PP768529	PP768564	PP768599
	JZB1556-3-2	Daoxianghu Park	PP709492	PP768460	PP768495	PP768530	PP768565	PP768600
	JZB1562-3-4	Qianlingshan Park	PP709493	PP768461	PP768496	PP768531	PP768566	PP768601
	JZB1492-6-1	Xishan Park	PP709494	PP768462	PP768497	PP768532	PP768567	PP768602
	JZB1372-3-5	Wufu Park	PP709495	PP768463	PP768498	PP768533	PP768568	PP768603
*C. aenigma*	JZB1040-11-2	Xiangshan Park	PP709496	PP768464	PP768499	PP768534	PP768569	PP768604
	JZB1241-3-8	Baiwangshan Park	PP709497	PP768465	PP768500	PP768535	PP768570	PP768605
	JZB1553-1-4	Banjing Road	PP709498	PP768466	PP768501	PP768536	PP768571	PP768606
	JZB1557-1-2	Daoxianghu Park	PP709499	PP768467	PP768502	PP768537	PP768572	PP768607
	JZB1562-3-1	Qianlingshan Park	PP709500	PP768468	PP768503	PP768538	PP768573	PP768608
	JZB1492-3-2	Xishan Park	PP709501	PP768469	PP768504	PP768539	PP768574	PP768609
*C. truncatum*	JZB1241-2-3	Baiwangshan Park	PP709502	PP768470	PP768505	PP768540	PP768575	PP768610
	JZB1125-3-6	Shuguang Park	PP709503	PP768471	PP768506	PP768541	PP768576	PP768611
	JZB1564-1-2	Qianlingshan Park	PP709504	PP768472	PP768507	PP768542	PP768577	PP768612
	JZB1372-4-1	Wufu Park	PP709505	PP768473	PP768508	PP768543	PP768578	PP768613
*C. subacidae*	JZB1241-2-2	Baiwangshan Park	PP709506	PP768474	PP768509	PP768544	PP768579	PP768614
	JZB1490-1-4	Xishan Park	PP709507	PP768475	PP768510	PP768545	PP768580	PP768615
*C. sojae*	JZB1124-3-1	Shuguang Park	PP709508	PP768476	PP768511	PP768546	PP768581	PP768616
	JZB1552-2-2	Banjing Road	PP709509	PP768477	PP768512	PP768547	PP768582	PP768617

For phylogenetic analysis, additional reference sequences were selected based on related studies on *Colletotrichum* species (Weir et al., 2012; Guarnaccia et al., 2017) and retrieved from GenBank. Individual gene datasets of representative isolates were aligned using MAFFT v. 7 (https://mafft.cbrc.jp/alignment/server/) and adjusted manually with BioEdit v. 7.0.9.0 where necessary. The maximum parsimony analyses (MP) were performed based on the multi-loci alignment using PAUP v. 4.0b10. The analysis involved running 1000 replicates of a heuristic search, which utilized random sequence addition for initial tree construction followed by tree bisection reconnection branch swapping (Swofford, 2002). Bayesian inference analysis was conducted using MrBayes 3.1.2. The phylogenetic trees were visualized via TreeviewX v. 0.5.0.

### 2.3 Morphological characterization

For morphological characterization, mycelial discs from growing edge of the fungal cultures were transferred to fresh PDA plates and incubated at 28°C for 5 days (Cai et al., 2009). Appressoria was produced by dropping 50 μL conidial suspension (10^6^ conidia/mL) on a concavity slide containing moistened filter papers with distilled sterile water, and then incubating at 28°C in the dark for 48 h (Weir et al., 2012). The shape, color and size of conidia (n=40) and appressoria (n=40) for each test *Colletotrichum* isolate were observed by light microscopy, and their dimensions were examined using an Axioscope 5 microscope (Carl Zeiss Microscopy, Germany). Mycelial growth rate of the representative *Colletotrichum* isolates was calculated by incubating the fresh mycelia blocks on new plates at 28°C with a photoperiod of 12 h/12 h for 5 days.

### 2.4 Pathogenicity test

*Colletotrichum* isolates representing different sampling sites or belonging to different *Colletotrichum* species were selected to conduct the pathogenicity test using mycelial plug method. Healthy leaves of *H. macrophylla* variety “Wujinxia” were collected, surface sterilized with 70% ethanol, washed three times with sterile distilled water, and then air dried on a sterilized tissue paper. Ten leaves per isolate with three replications were wounded by pin-pricking on both sides of the midrib with a sterilized needle, and 7-mm-diameter mycelia discs of 5-day-old cultures were inoculated, agar blocks without fungi were inoculated as control. Unwounded leaves were inoculated in the same way as described above. All the leaves were placed within a plastic box containing sterile water-soaked filter paper. The box was covered with plastic film and maintained in a growth chamber under condition of 85% relative humidity, a temperature of 28°C, and a 12/12 h light/dark photoperiod. Symptom development and lesion diameters on leaves were examined 7 days post inoculation. The fungus was re-isolated from lesions and recognized by integrated methods of morphological and molecular characteristics in order to fulfill Koch’s postulates.

### 2.5 Fungicide sensitivity of dominant *Colletotrichum* species

Representative isolates from the dominant *Colletotrichum* species, namely *C. gloeosporioides*, *C. fructicola*, and *C. aenigma* were selected and their sensitivities to three DMIs fungicides were tested using mycelial growth rate method (Zhang et al., 2020; Kim et al., 2020). The fungicides including prochloraz, difenoconazole, and tebuconazole were dissolved and adjusted to a concentration of 10 mg/ml as the stock solution. Each fungicide was prepared separately and the stock solutions were serially diluted as follows: prochloraz (0.005, 0.01, 0.02, 0.04, 0.08, 0.1 mg/mL), difenoconazole (0.005, 0.01, 0.05, 0.1, 0.5, 1, 5 mg/mL), tebuconazole (0.01, 0.05, 0.1, 0.5, 1, 5, 10 mg/mL), and then added to the sterilized PDA (approximately 50°C) at a ratio of 1:1000 (**Supplementary Table S2**). The margin of 5-day-old culture was used to produce 5-mm-diameter mycelial discs, which were then placed at the center of PDA plates with varying concentrations of fungicides. The diameter of each colony was measured in two perpendicular directions, after incubated at 28°C in the dark for 7 days. The percentage inhibition of mycelial growth for each *Colletotrichum* isolate at each test concentration (I) was also calculated as the difference between the radial growth of nonamended control (C) and the radial growth of each test concentration (T) as follows: I (%) = (C-T)/C×100. Each treatment was tested three times, with three plates for each replication. The EC_50_ values of the fungicides were calculated and displayed as the mean values derived from 12, 9, and 6 representative isolates of *C. gloeosporioides*, *C. fructicola*, and *C. aenigma*, respectively.

### 2.6 Statistical analysis

All the data were expressed as mean ± standard deviation of three replications unless otherwise mentioned. Significance of the differences (*P* < 0.05) was evaluated by one-way analysis of the variance (ANOVA) using the SPSS v21 software. The EC_50_ values were calculated by linear regression of the probit-transformed relative inhibition value on the log10- transformed fungicide concentration using the statistical algorithms.

## 3 Results

### 3.1 Disease survey and prevalence of *Colletotrichum* species

**Figure 1:**
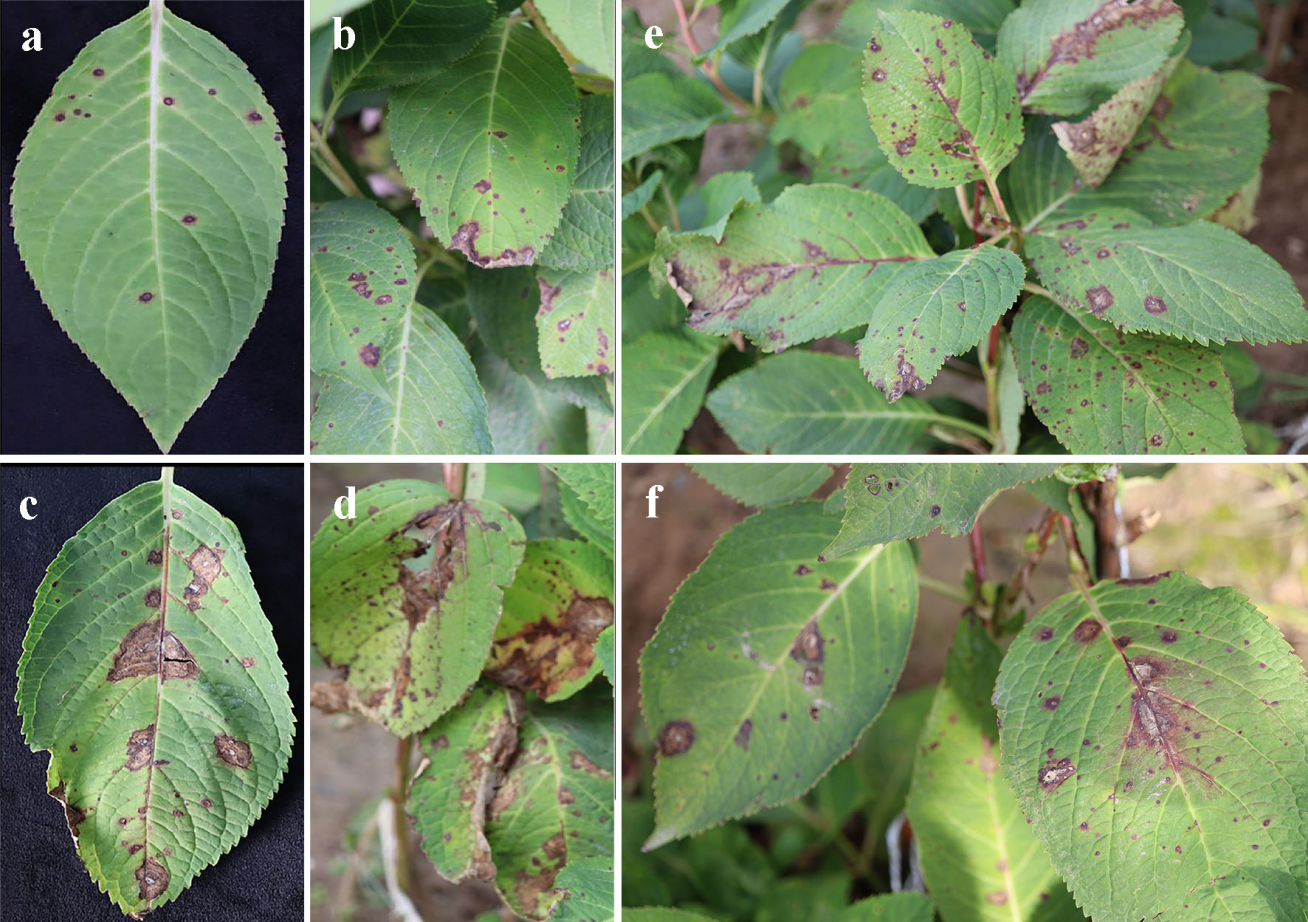
Typical symptoms of anthracnose on the leaves of *Hydrangea macrophylla* in Beijing, China. **(A, B)**. Symptoms of small spots on the leaves; **(C, D)**. Coalesce of brown necrotic lesions on the leaves; **(E, F)**. Anthracnose symptoms on the whole plants.

During the investigation of *H. macrophylla* foliar disease from 2021-2023, severe anthracnose symptoms were observed with disease incidence ranging from 23.3%-56.7% in nine parks across Beijing, China. The disease was first observed on newly emerged leaves of *H. macrophylla*, the infection quickly spread to the around plants in the late growing season. Typical symptoms were initially manifested as tiny purplish-red spots, approximately the size of pinheads with a yellow halo, which subsequently transitioned to light brown or grayish white with brown margins. As the symptoms advanced, these spots ultimately enlarged and merged, resulting in the formation of extensive necrotic regions (**Figure 1**). A total of 154 monosporic fungal isolates were recovered from symptomatic *H. macrophylla* leaves. In addition, certain species belonging to other genera like *Pythium*, *Alternaria*, and *Fusarium* were also detected during the isolation process but not shown in this study.

Based on morphology and rDNA-ITS sequence data, the remained 114 isolates resembling *Colletotrichum* were primarily assigned to three groups, *C. gloeosporioides* species complex (106 isolates), *C. truncatum* species complex (6 isolates), C. *orchidearum* species complex (2 isolates). Further identification based on sequence analysis of six gene loci indicated that, *C. gloeosporioides* was the most prevalent species (65 isolates, 57.0%) associated with *H. macrophylla* anthracnose, followed by *C. fructicola* (33 isolates, 28.9%), *C. aenigma* (8 isolates, 7.0%), *C. truncatum* (4 isolates, 3.5%), *C. subacidae* and *C. sojae* (2 isolates, 1.8% each). Among which, *C. gloeosporioides* was the prevalent species in all the parks, *C. subacidae* was only found in Xishan and Baiwang parks, while *C. sojae* was only detected in Shuguang Park and Banjing Road (**Table 1**; **Supplementary Figure S1**).

### 3.2 Multi-loci phylogenetic analysis

**Figure 2:**
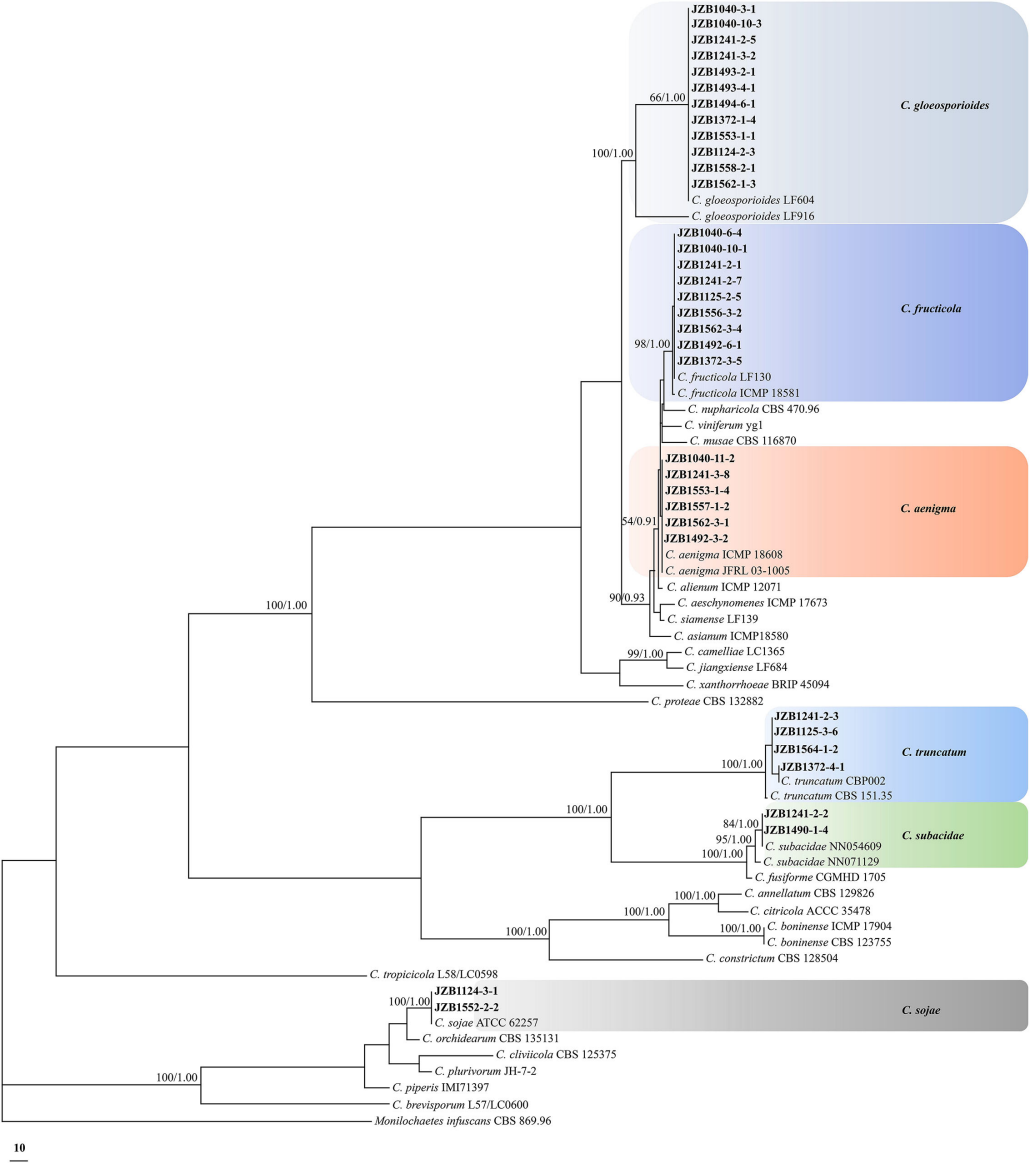
Phylogenetic tree based on the concatenated sequences of rDNA-ITS, *ACT*, *TUB2*, *CAL*, *CHS-1*, and *GAPDH* genomic regions of 35 representative *Colletotrichum* isolates obtained from *Hydrangea macrophylla* in Beijing, China. The species *Monilochaetes infuscans* CBS 869.96 was selected as an outgroup. Maximum likelihood boot strap support values (ML≥50) and Bayesian posterior probability values (PP≥0.90) were shown at the nodes, respectively. Colored blocks indicated clades containing isolates from different *Colletotrichum* species in this study.

A total of 35 representative *Colletotrichum* isolates from different sampling sites or belonging to different species were further subjected to multi-loci phylogenetic analysis with concatenated datasets of rDNA-ITS, *ACT*, *TUB2*, *CAL*, *CHS-1* and *GAPDH* sequences (**Figure 2**; **Table 2**). Phylogenetic analysis showed that the present *Colletotrichum* isolates from *H. macrophylla* anthracnose clearly clustered into three clades with *Monilochaetes infuscans* CBS 869.96 included as the outgroup (**Supplementary Table S3**). Among which, twelve isolates within the *C*. *gloeosporioides* species complex grouped together to formed a clade with the ex-type isolate of *C*. *gloeosporioides* LF604, nine isolates clustered with ex-type isolate of *C*. *fructicola* LF130, while the remaining six isolates constituted a distinct clade along with the ex-type isolate of *C*. *aenigma* ICMP18608 and JFRL03-1005. For phylogenetic analysis of the *C. truncatum* species complex, four isolates were grouped together with the ex-type isolates of *C. truncatum* CBP002, while two isolates formed a clade in conjunction with *C. subacidae* NN054609. In addition, two *Colletotrichum* isolates from *C. orchidearum* complex were clustered with *C. sojae* ATCC62257.

### 3.3 Morphological characterization

**Figure 3:**
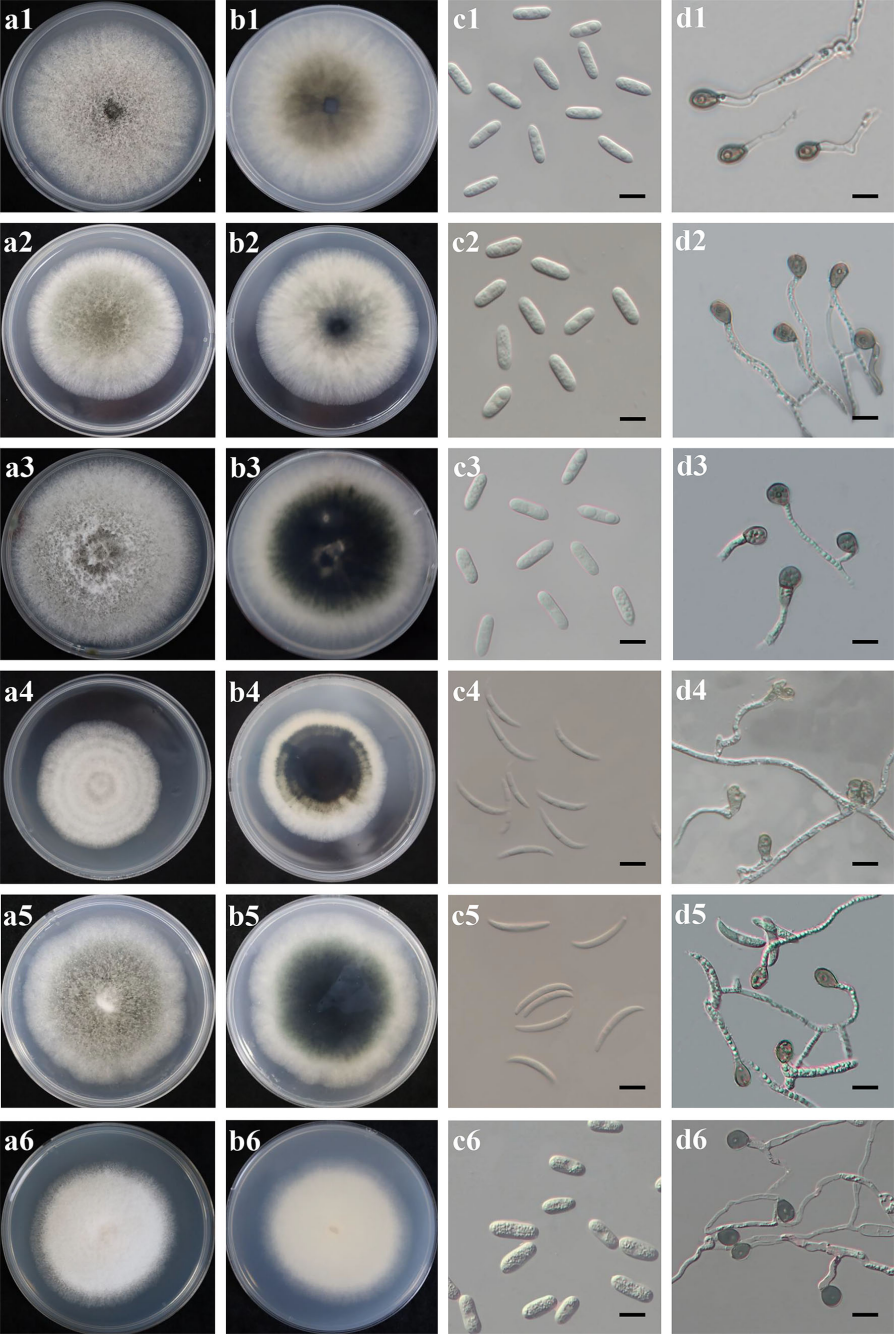
Culture characteristics and microscopic features of six *Colletotrichum* species obtained from *Hydrangea macrophylla* in Beijing, China. Each species was represented in four pictures **(A-D)**. **(A, B)**. Upper and reverse view of colony on potato dextrose agar at 28°C; **(C)**. Conidia, scale bars = 20 µm; **(D)**. Appressoria, scale bars = 20 µm. Plates 1-6 referred to *C. gloeosporides* JZB1040-3-1, *C. fructicola* JZB1241-2-7, *C. aenigma* JZB1040-11-2, *C. truncatum* JZB1564-1-2, *C.subacidae* JZB1490-1-4, and *C. sojae* JZB1124-3-1, respectively.

Distinct morphological features including colony, conidia and appressoria of 35 representative *Colletotrichum* isolates (**Table 2**) were observed for each identified *Colletotrichum* species after 7 days incubation on PDA (**Figure 3**). Most isolates in *C. gloeosporioides* species complex developed greyish white to pale grey colonies, while the reverse sides of *C. fructicola* and *C. aenigma* were grayish green to olivaceous grey with white margin. The conidia were all cylindrical with obtuse to slightly rounded ends. Appressoria were pale brown to dark brown, subglobose or ellipsoid, and rarely irregular. The *C. truncatum* and *C. orchidearum* species complex were easily distinguishable from *C. gloeosporioides* species complex in terms of conidia or appressoria shape (**Table 3**). Conidia of *C. truncatum* was crescent-shaped, smooth-walled, and slightly curved with parallel walls. Conidia of *C. subacidae* was slightly curved, acute apex, the central part was almost straight with parallel walls. The conidia of *C. sojae* were cylindrical with obtuse to slightly rounded ends, and their appressoria were dark brown, oval or bullet-shaped. There was a considerable variation in mycelial growth rate among the representative isolates belonging to different *Colletotrichum* species (**Table 3**). The average mycelial growth rate of *C. aenigma* reached 12.6 ± 0.4 mm/d followed by *C. gloeosporioides* and *C. fructicola*, while the growth rate of *C. sojae* was only 7.1 ± 0.3 mm/d.

**Table 3:** Morphological data of six *Colletotrichum* species associated with *Hydrangea macrophylla* anthracnose in Beijing, China.

Species	Growth rate (mm/day)	Colony appearance	Conidia (n=40)			Appressoria (n=40)		
			Length (μm)	Width (μm)	Shape	Length (μm)	Width (μm)	Shape
*C. gloeosporoides* (12 isolates)	11.9 ± 0.4	dense aerial mycelia, greyish white to pale gray, reverse dark brown with pale white margins	13.44-18.51 16.96 ± 1.37	4.54-6.30 5.30 ± 0.81	hyaline, aseptate, cylindrical with obtuse to slightly rounded ends	8.48-12.17 10.15 ± 0.81	6.14-9.51 7.24 ± 0.67	dark brown, ovoid to subglobose, slightly irregular, pear shaped
*C. fructicola* (9 isolates)	10.8 ± 0.5	dark grey with white halo edges, reverse grayish green in center	12.46-19.14 15.45 ± 2.13	4.52-7.49 5.90 ± 0.62	hyaline, aseptate, cylindrical with obtuse to slightly rounded ends	8.49-11.15 9.32 ± 1.25	6.53-10.56 7.18 ± 0.63	brown to dark black, ovoid to subglobose, lightly irregular
*C. aenigma* (6 isolates)	12.6 ± 0.4	dark gray, reverse olivaceous grey with white margin	12.74-16.79 15.37 ± 0.85	4.56-7.32 5.71 ± 0.43	hyaline, aseptate, cylindrical with broadly rounded ends	8.35-13.92 10.71 ± 0.25	5.85-8.56 6.48 ± 0.18	dark brown, ovoid to ellipsoid
*C. truncatum* (4 isolates)	8.4 ± 0.4	pale grey, reverse olivaceous to dark brown with white margins and gray-black strips	15.85-25.39 19.35 ± 1.87	3.44-4.32 3.89 ± 0.41	hyaline, aseptate, crescent-shaped, slightly curved with parallel walls	4.69-11.20 7.81 ± 1.20	4.10-7.05 5.46 ± 0.53	light brown to dark brown, ovoid to ellipsoidal, slightly irregular
*C. subacidae* (2 isolates)	8.9 ± 0.6	flat with undulate edge, smoke grey with white margin, reverse greenish grey	21.25-30.14 26.33 ± 2.35	2.56-4.28 3.15 ± 0.37	hyaline, aseptate, smooth-walled, slightly curved, acute apex	10.13-23.05 15.54 ± 4.42	5.10-7.34 6.25 ± 1.06	brown, ovoid to subcylindrical, rarely irregular
*C. sojae* (2 isolates)	7.1 ± 0.3	offwhite or light gray, reverse pale white to light orange	14.23-19.50 16.54 ± 1.82	4.05-5.93 4.97 ± 0.34	hyaline, aseptate, cylindrical with obtuse to slightly rounded ends	11.55-21.86 16.75 ± 2.86	5.31-8.97 7.20 ± 1.43	dark brown, oval, bullet-shaped or irregular

### 3.4 Pathogenicity test

**Figure 4:**
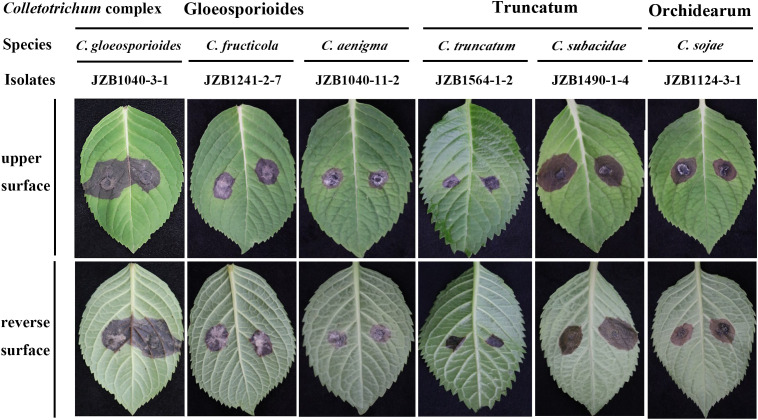
Symptoms of *Hydrangea macrophylla* leaves induced by inoculation of representative isolates from six *Colletotrichum* species under wounded conditions. The inoculation was conducted by dropping 5 mm mycelia disks of representative isolates on the detached leaves of *H. macrophylla*. Each leaf was wounded by pin-pricking with a sterilized needle. Control leaves were treated with sterilized agar blocks with the same size. The lesions on leaves were photographed 7 days post inoculation.

Pathogenicity test demonstrated that the 35 representative *Colletotrichum* isolates from different sampling sites or belonging to different species (**Table 2**) exhibited varying degrees of aggression on *H. macrophylla* leaves. Seven days after inoculation, all the tested isolates caused symptoms on the wounded leaves. The symptoms mainly manifested as dark brown or brownish, irregular lesions with yellow halos around their peripher on the surface of leaves, consistent with the symptoms observed in field (**Figure 4**). No lesions were induced in the control leaves inoculated with sterile PDA discs. Notably, certain species such as *C. gloeosporioides* JZB1040-3-1, *C. subacidae* JZB1490-1-4 exhibited the highest level of aggressiveness among the tested isolates. In contrast, *C. truncatum* JZB1564-1-2 produced small necrotic lesions. The remaining isolates had an intermediate level of aggressiveness (**Supplementary Figure S2**). To fulfill Koch’s postulates, the *Colletotrichum* species were re-isolated from the lesions of inoculated leaves and identified based on integrated analysis of morphological characteristics and multi-loci sequencing data. The re-obtained isolates matched well with the original ones that were used for inoculation. Further analysis showed that lesions on the wounded leaves were much larger than those on the unwounded leaves, indicating that wound is a crucial prerequisite for the occurrence of *H. macrophylla* anthracnose.

### 3.5 Fungicide sensitivity of dominant *Colletotrichum* species

A total of 27 representative isolates from the three dominant *Colletotrichum* species, namely *C. gloeosporioides*, *C. fructicola*, and *C. aenigma* (**Supplementary Table S4**), were chosen to determine their sensitivities to fungicides using the mycelial growth method (**Supplementary Figure S3**). Regarding the relative fungicide sensitivity of individual *Colletotrichum* species, we found that *C. gloeosporioides*, *C. fructicola* and *C. aenigma* exhibited greater sensitivity to prochloraz, since their mean EC_50_ values were only 0.062, 0.033, and 0.023 µg/ml. *Colletotrichum fructicola* exhibited significantly lower EC_50_ values against prochloraz than difenoconazole and tebuconazole. Among the three species, there were no significant differences in the EC_50_ values respect to difenoconazole and tebuconazole (**Table 4**). The fungicide sensitivities also varied among isolates within the same species. For prochloraz, the EC_50_ values of *C. gloeosporioides* spanned from 0.004 to 0.219 µg/ml, while those of *C. fructicola* ranged from 0.003 to 0.074 µg/ml and those of *C. aenigma* ranged from 0.007 to 0.064 µg/ml. Some isolates within *C. gloeosporioides* demonstrated an obviously reduced sensitivity to difenoconazole and tebuconazole, with EC_50_ values reaching 3.100 and 3.677 µg/ml, respectively (**Supplementary Table S4**).

**Table 4:** Fungicides sensitivity of three dominant *Colletotrichum* species associated with *Hydrangea macrophylla* anthracnose in Beijing, China.

Fungicides	*C. gloeosporioides* (12 isolates)		*C. fructicola* (9 isolates)		*C. aenigma* (6 isolates)	
	EC_50_ (μg/ml)		EC_50_ (μg/ml)		EC_50_ (μg/ml)	
	range	mean ± SD	range	mean ± SD	range	mean ± SD
Prochloraz	0.004-0.219	0.062 ± 0.066 a	0.003-0.074	0.033 ± 0.026 b	0.007-0.064	0.023 ± 0.022 a
Difenoconazole	0.010-3.100	0.749 ± 0.905 a	0.029-0.742	0.345 ± 0.298 a	0.039-0.779	0.215 ± 0.291 a
Tebuconazole	0.045-3.677	0.431 ± 1.039 a	0.042-0.614	0.153 ± 0.187 a	0.039-0.185	0.122 ± 0.057 a

Means followed by different letters indicate significant differences within each species based on ANOVA (*P*<0.05).

## 4 Discussion

Plant anthracnose can be induced by numerous *Colletotrichum* species. Extensive host range and wide geographic distribution of *Colletotrichum* species might be ascribed to their enhanced genetic diversity to adapt to various environmental conditions. Series studies have been conducted on the pathogen composition of *Colletotrichum* associated with plant anthracnose (Guarnaccia et al., 2017; Zhou et al., 2023). In this study, based on morphological observation and phylogenetic analysis, the *Colletotrichum* isolates associated with *H*. *macrophylla* anthracnose were identified as belonging to six species including *C. gloeosporioides*, *C. fructicola*, *C. aenigae*, *C. truncatum*, *C. subacidae* and *C. sojae*. This is the first report of the later four *Colletotrichum* species causing *H. macrophylla* anthracnose worldwide including China.

Traditionally, the species delimitation in *Colletotrichum* was mainly based on host range and morphological characteristic, such as the shape, color, dimension of colonies, conidia, and appressoria (Cai et al., 2009). In recent years, the number of new *Colletotrichum* species has increased dramatically with the development of molecular technologies, as the formerly recognized species have been dissected into species complexes, each of which encompasses numerous phylogenetically distinct species. For example, based on the combined sequence data of six gene loci, all the *Colletotrichum* isolates associated with strawberry anthracnose were grouped into three clades, namely *C*. *siamense*, *C*. *fructicola*, and *C*. *aenigma*, which were formerly part of *C. gloeosporioides* species complex (Zhang et al., 2020). Among the multiple genomic regions, *TUB2* could discriminate all species within the *C. orchidearum* complex. *CHS-1* can assist in distinguishing and corroborating recently diverged species, and thus serves as an informative marker for *Colletotrichum* species complexes (Vieira et al., 2020). In this study, phylogenetic analysis derived from the molecular data of ITS, *ACT*, *TUB2, CAL*, *CHS-1*, and *GAPDH* gene sequences attributed all the *Colletotrichum* isolates into six species, which was in full accordance with the results of the morphological groupings.

*Colletotrichum gloeosporioide*, *C. fructicola*, and *C. aenigma* within the *C. gloeosporioides* species complex are globally distributed and possess a wide variety of host species (Zhou et al., 2023). Besides, *C. truncatum* has also been cited as a pathogen of many economically important plants worldwide, such as papaya (Aktaruzzaman et al., 2018), watermelon (Guo et al., 2022). *Colletotrichum subacidae* were obtained from the diseased stem of *Asparagus officinalison* and the leaf petiole of *Ailanthus altissima* in China (Liu et al., 2022). *Colletotrichum sojae* was described as causal agent of anthracnose on pepper (Zhang et al., 2022) and American Ginseng (Guan et al., 2021) in China. This study demonstrated that all the six *Colletotrichum* species were capable of infecting leaves of *H. macrophylla* with *C. gloeosporioides* being the most prevalent species. Previous results indicated that wounding can break the quiescent infection and enhance the infectivity of *Colletotrichum* species, thereby resulting in a more rapid progression in wounded leaves (Jiang et al., 2014). Many *Colletotrichum* isolates cause obvious lesions on leaves under wounded conditions, but not under unwounded conditions (Fu et al., 2019). In this study, pathogenicity test of *Colletotrichum* species were conducted under both wounded and unwounded conditions, it was found that wound is the essential condition for the occurrence of *H*. *macrophylla* anthracnose. Therefore, wounds when transplanting or pruning should be avoided in actual production so as to prevent pathogen infection and disease transmission.

Prochloraz, difenoconazole, and tebuconazole are the DMI fungicides that have been employed in the management of anthracnose in China (Zhang et al., 2017). In this study, three DMI type fungicides were used to assess their inhibitory activity against the dominant *Colletotrichum* species. Three species C. *glosporioides*, *C. fructicola*, and *C. aenigma* differed in sensitivity to certain fungicides with no variation. Among which, the effect of prochloraz was superior to that of difenoconazole and tebuconazole. *Colletotrichum glosporioides* exhibited reduced sensitivity to difenoconazole and tebuconazole compared with the isolates from the other two species, with three being species all from the *C. gloeosporioides* complex. DMI fungicides are classified as moderately risk in terms of the development of fungicide resistance, moreover, DMI-resistant strains have been detected on numerous crops (Shi et al., 2020). Reduced sensitivity or even resistance to tebuconazole have been reported on *C. gloeosporioides*, the causal agent of anthracnose on walnut (Wang et al., 2020) and chili (Wei et al., 2020). Therefore, attention should be paid to the sensitivity of the dominant *Colletotrichum* species of *H. macrophylla* to such fungicides, to prevent the emergence of resistance. In addition, the combined or alternative application of fungicides with different action modes should be adopted to reduce the risk of resistance (Wang et al., 2020).

Better understanding of species distribution and individual characteristics of the *Colletotrichum* species associated with plant anthracnose is critical for development of disease management plans. This study represents the first comprehensive investigation of *Colletotrichum* species occurring on *H. macrophylla* anthracnose in Beijing, China. The knowledge acquired and the diversity of *Colletotrichum* species in *H. macrophylla* gathered provides a useful clue for resistant-germplasm selection and disease management. Future work may focus on the pathogenic mechanism of the dominant *Colletotrichum* species responsible for *H. macrophylla* anthracnose, as well as fungicide resistance risk assessment or resistance gene identification.

## 5 Conclusions

Here we provided the first detailed investigation of *Colletotrichum* species associated with anthracnose of *Hydrangea macrophylla* in Beijing, China. A total number of 114 *Colletotrichum* isolates belonging to six *Colletotrichum* species were characterized, and three species in *C. gloeosporioides* species complex were confirmed as the dominant species. We also demonstrated, for the first time, that *C. aenigae*, *C. truncatum*, *C. subacidae* and *C. sojae* were responsible for *H. macrophylla* in Beijing, China. Our results disclosed that these *Colletotrichum* taxa were pathogenic to *H. macrophylla* with varied aggressiveness, wound was the crucial condition for pathogen infection. Additionally, fungicide sensitivity showed that the inhibition effect of prochloraz were superior to that of difenoconazole and tebuconazole. Overall, this study provides crucial information for the management of *H. macrophylla* anthracnose in Beijing, China.

## Data Availability

The datasets presented in this study can be found in online repositories. The names of the repository/repositories and accession number(s) can be found in the article/**Supplementary Material**.
