# Supplementary material for: Characterization and fungicides sensitivity of Colletotrichum species causing Hydrangea macrophylla anthracnose in Beijing, China
Source: Front Plant Sci. 2025 Jan 21;15:1504135. doi: 10.3389/fpls.2024.1504135 (PMC11790641; doi:10.3389/fpls.2024.1504135)
Supplement: Supplementary file 1 [file DataSheet2.docx]

**Supplementary materials**

**Supplementary Table S1** PCR primers used for molecular characterization of *Colletotrichum* isolates from *Hydrangea macrophylla* in Beijing, China

| Region | Primer | Sequence (5′-3′) | Tm (℃) | References |  |
| --- | --- | --- | --- | --- | --- |
| rDNA-ITS | ITS1 | TCCGTAGGTGAACCTGCGG | 56 | White et al., 1990 |  |
|  | ITS4 | TCCTCCGCTTATTGATATGC |  |  |  |
| actin (*ACT*) | ACT-512F | ATGTGCAAGGCCGGTTTCGC | 58 | Carbon and Kohn, 1999 |  |
|  | ACT-783R | TACGAGTCCTTCTGGCCCAT |  |  |  |
| β-tubulin (*TUB2*) | T1 | AACATGCGTGAGATTGTAAGT | 55 | Glass and Donaldson, 1995  O’Donnell and Cigelnik, 1997 |  |
|  | Bt2b | ACCCTCAGTGTAGTGACCCTTGGC |  |  |  |
| calmodulin (*CAL*) | CL1C | GAATTCAAGGAGGCCTTCTC | 55 | Weir et al., 2012 |  |
|  | CL2C | CTTCTGCATCATGAGCTGGAC |  |  |  |
| chitin synthase (*CHS-1*) | | CHS-79F | TGGGGCAAGGATGCCTGGAAGAAG | 58 | Carbone and Kohn, 1999 |
|  |  | CHS-354R | TGGAAGAACCATCTGTGAGAGTTG |  |  |
| glyceraldehyde-3-phosphate dehydrogenase (*GAPDH*) | GDF1 | GCCGTCAACGACCCCTTCATTGA | 60 | Templeton et al., 1992 |  |
|  | GDR1 | GGGTGGAGTCGTACTTGAGCATGT |  |  |  |

**Supplementary Table S2** Information of chemicals used for fungicides sensitivity assay

| Fungicides | Manufacturer | Type | Active ingredients content | Concentrations (µg/ml) |
| --- | --- | --- | --- | --- |
| Prochloraz | FMC (Suzhou) Crop Care | EW | 450 g/L | 0.005, 0.01, 0.02, 0.04, 0.08, 0.1 |
| Difenoconazole | Syngenta Nantong Crop Protection | WDG | 10% | 0.005, 0.01, 0.05, 0.1, 0.5, 1, 5 |
| Tebuconazole | Bayer Crop Science China | SC | 430 g/L | 0.01, 0.05, 0.1, 0.5, 1, 5, 10 |

Note: EW. emulsion in water; WDG. water dispersible granule; SC. suspension concentrate.

**Supplementary Table S3** Representative *Colletotrichum* isolates and their GenBank accession numbers used for phylogenetic analysis

| Species | Isolates numbers | GenBank accession number | | | | | |
| --- | --- | --- | --- | --- | --- | --- | --- |
|  |  | rDNA-ITS | *ACT* | *TUB2* | *CAL* | *CHS1* | *GAPDH* |
| *C. aenigma* | ICMP:18608, C1253.4 | JX010244 | JX009443 | JX010389 | JX009683 | JX009774 | JX010044 |
| *C. aenigma* | JFRL 03-1005, GT-51 | OQ600619 | OQ603370 | OQ603379 | OQ974177 | OQ189876 | OQ603373 |
| *C. alienum* | ICMP:12071, C824 | JX010251 | JX009572 | JX010411 | JX009654 | JX009882 | JX010028 |
| *C. aeschynomenes* | ICMP:17673, 3-1-3 | JX010176 | JX009483 | JX010392 | JX009721 | JX009799 | JX009930 |
| *C. annellatum* | CBS:129826 | JQ005222 | JQ005570 | JQ005656 | JQ005743 | JQ005396 | JQ005309 |
| *C. asianum* | ICMP:18580, C1315.4 | JX010196 | JX009584 | JX010406 | JX009727 | JX009867 | JX010053 |
| *C. brevisporum* | L57/LC0600, BCC 38876 | JN050238 | JN050216 | JN050244 | JN050222 | MZ799287 | JN050227 |
| *C. boninense* | ICMP:17904, MAFF305972 | JX010292 | JX009583 | HM585421 | JX009741 | HM582032 | JX009905 |
| *C. boninense* | CBS:123755 | JQ005153 | JQ005501 | JQ005588 | JQ005674 | JQ005327 | JQ005240 |
| *C. camelliae* | LC1365, HY1 | JN936983 | JN936971 | JN936977 | JN936974 | OQ506550 | JN936986 |
| *C. citricola* | ACCC 35478 | OR240824 | OR251089 | OR251096 | OR251103 | OR251075 | OR251068 |
| *C. cliviicola* | CBS 125375, LF774 | MH863509 | MG600939 | MG601000 | KJ954766 | MG600850 | MG600795 |
| *C. constrictum* | CBS:128504 | JQ005238 | JQ005586 | JQ005672 | JQ005759 | JQ005412 | JQ005325 |
| *C. fructicola* | LF130, HNLD-10 | KJ955083 | KJ954365 | KJ955232 | KJ954636 | MK675281 | KJ954784 |
| *C. fructicola* | ICMP:18581, C1315.3 | JX010165 | JX009501 | JX010405 | JX009676 | JX009866 | JX010033 |
| *C. fusiforme* | CGMHD 1705, 1704, 1622 | MN539248 | MN583276 | MN583303 | - | MN583284 | MN583292 |
| *C. gloeosporioides* | LF916, C20 | KJ955226 | KJ954493 | KJ955371 | KJ954777 | MF668114 | KJ954927 |
| *C. gloeosporioides* | LF604, CBS 273.51 | KJ955176 | KJ954450 | KJ955323 | KJ954728 | JX009903 | KJ954877 |
| *C. jiangxiense* | LF684, SYD-9 | KJ955198 | KJ954469 | KJ955345 | KJ954749 | OR472537 | KJ954899 |
| *C. musae* | CBS:116870 | JX010146 | JX009433 | JX010413 | JX009742 | JX009896 | JX010050 |
| *C. nupharicola* | CBS:470.96, ICMP:18187, C1275.25 | JX010187 | JX009437 | JX010398 | JX009663 | JX009835 | JX009972 |
| *C. orchidearum* | CBS 135131, SAUCC 1407 | MG600738 | MG600944 | MG601005 | KT362186 | MG600855 | MG600800 |
| *C. piperis* | IMI71397 | MG600760 | MG600964 | MG601027 | - | MG600867 | MG600820 |
| *C. plurivorum* | JH-7-2 | MW507147 | MW524860 | MW507372 | MW507369 | MW507370 | MW507371 |
| *C. proteae* | CBS 132882 | KC297079 | KC296940 | KC297101 | KC296960 | KC296986 | KC297009 |
| *C. siamense* | LF139, C1315.2 | KJ955087 | KJ954369 | KJ955236 | KJ954640 | JX009865 | KJ954788 |
| *C. sojae* | ATCC 62257, PCAHFY5 | KC110794 | KC110830 | KC110821 | OP750987 | MG600860 | KC110812 |
| *C. subacidae* | NN054609 | MZ595894 | MZ664192 | MZ674012 | - | MZ799310 | MZ664076 |
| *C. subacidae* | NN071129 | MZ595909 | MZ664207 | MZ674027 | - | MZ799311 | MZ664071 |
| *C. tropicicola* | L58/LC0598, BCC 38877 | JN050240 | JN050218 | JN050246 | JN050223 | MZ799279 | JN050229 |
| *C. truncatum* | CBP002 | KF030677 | KF158412 | KF240819 | KF114851 | KT778592 | KF300886 |
| *C. truncatum* | CBS:151.35 | GU227862 | GU227960 | GU228156 | KY856132 | GU228352 | GU228254 |
| *C. viniferum* | yg1, CGW01, DLL2-3-1 | JN412804 | JN412795 | JN412813 | LC684921 | OR043555 | JN412798 |
| *C. xanthorrhoeae* | BRIP:45094, C1271 | JX010261 | JX009478 | JX010448 | JX009653 | JX009823 | JX009927 |
| *Monilochaetes infuscans* | CBS 869.96 | JQ005780 | JQ005843 | JQ005864 | - | JQ005801 | JX546612 |

Note: CBS. Culture Collection of the Centralbureau Voor Schimmel-cultures, Fungal Biodiversity Centre, Utrecht, the Netherlands; ICMP. International Collection of Microorganisms from Plants, Auckland, New Zealand.

**Supplementary Table S4** Virulence regression equations and EC_50_ values of representative isolates from three dominant *Colletotrichum* species to the tested fungicides

| Fungicides | Isolates number | Species | Equation | R^2^ | EC_50_ (µg/ml) | Std. Error | F value |
| --- | --- | --- | --- | --- | --- | --- | --- |
| Prochloraz | JZB1040-3-1 | *C. gloeosporioides* | y=1.2493x+6.7163 | 0.9907 | 0.042 | 0.0702 | 310.0 |
| Prochloraz | JZB1040-10-3 | *C. gloeosporioides* | y=0.9707x+6.2325 | 0.9788 | 0.054 | 0.0848 | 128.3 |
| Prochloraz | JZB1241-2-5 | *C. gloeosporioides* | y=1.3975x+6.6732 | 0.9910 | 0.063 | 0.0728 | 361.8 |
| Prochloraz | JZB1241-3-2 | *C.gloeosporioides* | y=1.0086x+7.0698 | 0.9843 | 0.009 | 0.0637 | 250.5 |
| Prochloraz | JZB1493-2-1 | *C. gloeosporioides* | y=0.8701x+5.7358 | 0.9837 | 0.143 | 0.0629 | 187.5 |
| Prochloraz | JZB1493-4-1 | *C. gloeosporioides* | y=0.9219x+5.8902 | 0.9419 | 0.108 | 0.1310 | 48.6 |
| Prochloraz | JZB1494-6-1 | *C. gloeosporioides* | y=0.9813x+5.6477 | 0.9088 | 0.219 | 0.2154 | 20.2 |
| Prochloraz | JZB1372-1-4 | *C. gloeosporioides* | y=0.6794x+6.2401 | 0.9603 | 0.015 | 0.0810 | 68.9 |
| Prochloraz | JZB1553-1-1 | *C. gloeosporioides* | y=1.2665x+6.4673 | 0.9846 | 0.069 | 0.1138 | 120.4 |
| Prochloraz | JZB1124-2-3 | *C. gloeosporioides* | y=1.118x+7.4364 | 0.9141 | 0.007 | 0.1702 | 34.0 |
| Prochloraz | JZB1558-2-1 | *C. gloeosporioides* | y=0.6247x+6.5192 | 0.9743 | 0.004 | 0.0507 | 151.8 |
| Prochloraz | JZB1562-1-3 | *C. gloeosporioides* | y=1.1858x+7.4903 | 0.9706 | 0.008 | 0.1031 | 132.3 |
| Prochloraz | JZB1040-6-4 | *C. fructicola* | y=0.9259x+7.0272 | 0.9791 | 0.007 | 0.0676 | 187.7 |
| Prochloraz | JZB1040-10-1 | *C. fructicola* | y=1.2613x+6.6934 | 0.9943 | 0.045 | 0.0554 | 507.7 |
| Prochloraz | JZB1241-2-1 | *C. fructicola* | y=1.1164x+6.4136 | 0.9968 | 0.054 | 0.0336 | 1086.0 |
| Prochloraz | JZB1241-2-7 | *C. fructicola* | y=1.5221x+6.7215 | 0.9269 | 0.074 | 0.2426 | 38.6 |
| Prochloraz | JZB1125-2-5 | *C. fructicola* | y=1.1547x+6.4919 | 0.9949 | 0.051 | 0.0452 | 641.2 |
| Prochloraz | JZB1556-3-2 | *C. fructicola* | y=0.9704x+7.0928 | 0.9778 | 0.007 | 0.0866 | 122.9 |
| Prochloraz | JZB1562-3-4 | *C. fructicola* | y=1.0095x+6.9703 | 0.9105 | 0.011 | 0.2054 | 20.3 |
| Prochloraz | JZB1492-6-1 | *C. fructicola* | y=1.1258x+6.5427 | 0.9687 | 0.043 | 0.1133 | 97.0 |
| Prochloraz | JZB1372-3-5 | *C. fructicola* | y=0.7886x+7.0188 | 0.9660 | 0.003 | 0.0740 | 113.7 |
| Prochloraz | JZB1040-11-2 | *C. aenigma* | y=3.8104x+9.5584 | 0.9006 | 0.064 | 0.2108 | 143.9 |
| Prochloraz | JZB1241-3-8 | *C. aenigma* | y=1.5381x+7.2932 | 0.9302 | 0.032 | 0.2106 | 53.3 |
| Prochloraz | JZB1553-1-4 | *C. aenigma* | y=1.0423x+7.2622 | 0.9887 | 0.007 | 0.0557 | 350.9 |
| Prochloraz | JZB1557-1-2 | *C. aenigma* | y=0.8813x+6.5625 | 0.9858 | 0.017 | 0.2413 | 24.7 |
| Prochloraz | JZB1562-3-1 | *C. aenigma* | y=1.0414x+7.0848 | 0.9816 | 0.010 | 0.0714 | 212.9 |
| Prochloraz | JZB1492-3-2 | *C. aenigma* | y=1.1035x+7.2176 | 0.9615 | 0.010 | 0.1104 | 99.9 |
| Difenoconazole | JZB1040-3-1 | *C. gloeosporioides* | y=0.5805x+5.2186 | 0.9603 | 0.420 | 0.0412 | 240.7 |
| Difenoconazole | JZB1040-10-3 | *C. gloeosporioides* | y=0.5814x+5.0832 | 0.9957 | 0.719 | 0.0332 | 361.5 |
| Difenoconazole | JZB1241-2-5 | *C. gloeosporioides* | y=0.7825x+4.8943 | 0.9278 | 1.365 | 0.1002 | 73.9 |
| Difenoconazole | JZB1241-3-2 | *C.gloeosporioides* | y=0.6956x+6.3993 | 0.9667 | 0.010 | 0.0392 | 121.9 |
| Difenoconazole | JZB1493-2-1 | *C. gloeosporioides* | y=0.6764x+4.8978 | 0.9334 | 1.416 | 0.0777 | 92.2 |
| Difenoconazole | JZB1493-4-1 | *C. gloeosporioides* | y=0.5527x+5.0049 | 0.9337 | 0.980 | 0.0611 | 99.9 |
| Difenoconazole | JZB1494-6-1 | *C. gloeosporioides* | y=0.6741x+4.6688 | 0.9579 | 3.100 | 0.0417 | 319.8 |
| Difenoconazole | JZB1372-1-4 | *C. gloeosporioides* | y=0.5959x+5.5958 | 0.9655 | 0.100 | 0.0297 | 220.0 |
| Difenoconazole | JZB1553-1-1 | *C. gloeosporioides* | y= 0.673x+5.0796 | 0.9625 | 0.762 | 0.0474 | 244.6 |
| Difenoconazole | JZB1124-2-3 | *C. gloeosporioides* | y=0.789x+6.0743 | 0.9435 | 0.043 | 0.1115 | 50.1 |
| Difenoconazole | JZB1558-2-1 | *C. gloeosporioides* | y=0.794x+6.2756 | 0.9638 | 0.025 | 0.1350 | 37.1 |
| Difenoconazole | JZB1562-1-3 | *C. gloeosporioides* | y=0.7216x+5.9888 | 0.9740 | 0.043 | 0.0371 | 440.3 |
| Difenoconazole | JZB1040-6-4 | *C. fructicola* | y=0.8943x+6.1614 | 0.9977 | 0.036 | 0.0248 | 1306.0 |
| Difenoconazole | JZB1040-10-1 | *C. fructicola* | y=0.5764x+5.1189 | 0.9574 | 0.622 | 0.0398 | 255.6 |
| Difenoconazole | JZB1241-2-1 | *C. fructicola* | y=0.6x+5.1497 | 0.9577 | 0.563 | 0.0404 | 269.1 |
| Difenoconazole | JZB1241-2-7 | *C. fructicola* | y=0.672x+5.0869 | 0.9421 | 0.742 | 0.0645 | 132.4 |
| Difenoconazole | JZB1125-2-5 | *C. fructicola* | y=0.6584x+5.1681 | 0.9712 | 0.556 | 0.0371 | 379.6 |
| Difenoconazole | JZB1556-3-2 | *C. fructicola* | y=0.562x+5.6617 | 0.9755 | 0.066 | 0.0292 | 90.6 |
| Difenoconazole | JZB1562-3-4 | *C. fructicola* | y=0.4718x+5.7266 | 0.9287 | 0.029 | 0.0232 | 228.9 |
| Difenoconazole | JZB1492-6-1 | *C. fructicola* | y=0.5478x+5.1838 | 0.9856 | 0.462 | 0.0137 | 1923.0 |
| Difenoconazole | JZB1372-3-5 | *C. fructicola* | y=0.7784x+6.1526 | 0.9564 | 0.033 | 0.0989 | 70.1 |
| Difenoconazole | JZB1040-11-2 | *C. aenigma* | y=0.5586x+5.3099 | 0.9708 | 0.279 | 0.0282 | 476.4 |
| Difenoconazole | JZB1241-3-8 | *C. aenigma* | y=0.7878x+5.0856 | 0.9743 | 0.779 | 0.047 | 338.0 |
| Difenoconazole | JZB1553-1-4 | *C. aenigma* | y=0.7521x+5.9526 | 0.9837 | 0.054 | 0.0558 | 181.6 |
| Difenoconazole | JZB1557-1-2 | *C. aenigma* | y=0.7989x+5.8133 | 0.9428 | 0.096 | 0.1537 | 28.8 |
| Difenoconazole | JZB1562-3-1 | *C. aenigma* | y=0.7258x+6.0163 | 0.9837 | 0.040 | 0.1197 | 38.8 |
| Difenoconazole | JZB1492-3-2 | *C. aenigma* | y=0.9025x+6.2667 | 0.9868 | 0.039 | 0.0781 | 150.8 |
| Tebuconazole | JZB1040-3-1 | *C. gloeosporioides* | y=0.5501x+5.0818 | 0.9641 | 0.710 | 0.0531 | 107.4 |
| Tebuconazole | JZB1040-10-3 | *C. gloeosporioides* | y=0.8375x+6.1276 | 0.9531 | 0.045 | 0.1073 | 60.9 |
| Tebuconazole | JZB1241-2-5 | *C. gloeosporioides* | y=0.8659x+6.0179 | 0.9561 | 0.067 | 0.1070 | 68.6 |
| Tebuconazole | JZB1241-3-2 | *C.gloeosporioides* | y=0.9183x+6.0829 | 0.9863 | 0.066 | 0.0540 | 288.7 |
| Tebuconazole | JZB1493-2-1 | *C. gloeosporioides* | y= 0.595x+5.6563 | 0.9682 | 0.079 | 0.0623 | 91.3 |
| Tebuconazole | JZB1493-4-1 | *C. gloeosporioides* | y=0.7558x+5.6134 | 0.9512 | 0.154 | 0.0856 | 77.9 |
| Tebuconazole | JZB1494-6-1 | *C. gloeosporioides* | y=0.6602x+5.8183 | 0.9904 | 0.058 | 0.0286 | 532.7 |
| Tebuconazole | JZB1372-1-4 | *C. gloeosporioides* | y=0.7178x+5.6315 | 0.9748 | 0.132 | 0.0576 | 154.5 |
| Tebuconazole | JZB1553-1-1 | *C. gloeosporioides* | y=0.5422x+4.6934 | 0.9856 | 3.677 | 0.0389 | 90.3 |
| Tebuconazole | JZB1124-2-3 | *C. gloeosporioides* | y=0.8836x+6.064 | 0.9773 | 0.062 | 0.0674 | 172.1 |
| Tebuconazole | JZB1558-2-1 | *C. gloeosporioides* | y=1.0698x+6.4339 | 0.9583 | 0.046 | 0.1400 | 72.8 |
| Tebuconazole | JZB1562-1-3 | *C. gloeosporioides* | y=0.6997x+5.7715 | 0.9691 | 0.079 | 0.0818 | 60.3 |
| Tebuconazole | JZB1040-6-4 | *C. fructicola* | y=1.0465x+6.2677 | 0.9823 | 0.061 | 0.0703 | 221.7 |
| Tebuconazole | JZB1040-10-1 | *C. fructicola* | y=1.1837x+5.655 | 0.9832 | 0.280 | 0.1003 | 148.0 |
| Tebuconazole | JZB1241-2-1 | *C. fructicola* | y=0.8658x+6.1961 | 0.9337 | 0.042 | 0.1332 | 42.3 |
| Tebuconazole | JZB1241-2-7 | *C. fructicola* | y=0.8546x+5.8907 | 0.9214 | 0.091 | 0.1248 | 46.9 |
| Tebuconazole | JZB1125-2-5 | *C. fructicola* | y=0.6624x+5.738 | 0.9696 | 0.077 | 0.0677 | 95.7 |
| Tebuconazole | JZB1556-3-2 | *C. fructicola* | y=0.8018x+5.8516 | 0.9623 | 0.087 | 0.0916 | 76.6 |
| Tebuconazole | JZB1562-3-4 | *C. fructicola* | y=0.6684x+5.1417 | 0.9619 | 0.614 | 0.0665 | 100.9 |
| Tebuconazole | JZB1492-6-1 | *C. fructicola* | y=0.666x+5.8293 | 0.9842 | 0.057 | 0.0665 | 100.9 |
| Tebuconazole | JZB1372-3-5 | *C. fructicola* | y=0.8476x+5.9915 | 0.9796 | 0.067 | 0.0954 | 81.8 |
| Tebuconazole | JZB1040-11-2 | *C. aenigma* | y=0.6297x+5.4616 | 0.9508 | 0.185 | 0.0827 | 57.9 |
| Tebuconazole | JZB1241-3-8 | *C. aenigma* | y=0.9118x+6.0186 | 0.9859 | 0.076 | 0.0546 | 279.4 |
| Tebuconazole | JZB1553-1-4 | *C. aenigma* | y=0.7885x+5.6989 | 0.9657 | 0.130 | 0.0743 | 112.6 |
| Tebuconazole | JZB1557-1-2 | *C. aenigma* | y=0.6973x+5.5192 | 0.9786 | 0.180 | 0.0631 | 143.9 |
| Tebuconazole | JZB1562-3-1 | *C. aenigma* | y=0.7995x+5.726 | 0.9695 | 0.124 | 0.1065 | 50.9 |
| Tebuconazole | JZB1492-3-2 | *C. aenigma* | y=0.9025x+6.2667 | 0.9868 | 0.039 | 0.0409 | 391.4 |


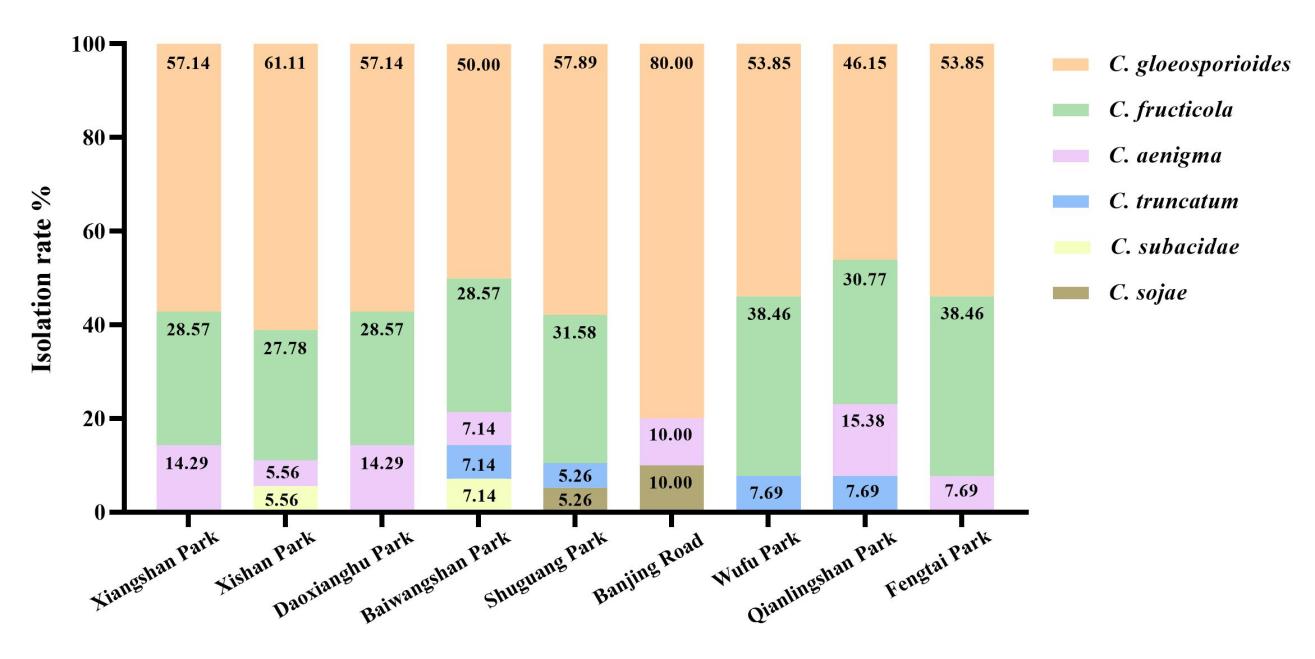


**Supplementary Figure S1** Isolation rate (%) of *Colletotrichum* species from anthracnose leaves of *Hydrangea macrophylla* in nine parks of Beijing, China


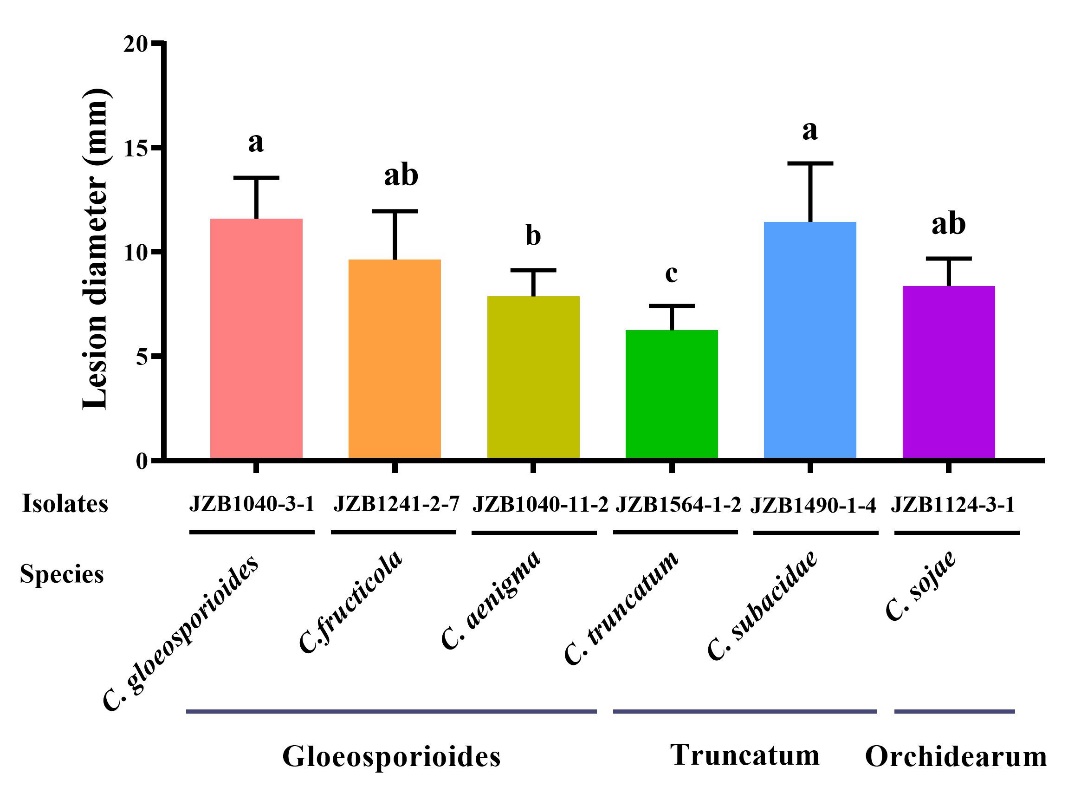


**Supplementary Figure S2** Lesion sizes on wound leaves of *Hydrangea macrophylla* inoculated with mycelial discs of the representative isolates from six *Colletotrichum* species. Bars with different lowercase letters indicated significant differences (one-way ANOVA, *P*< 0.05)

**
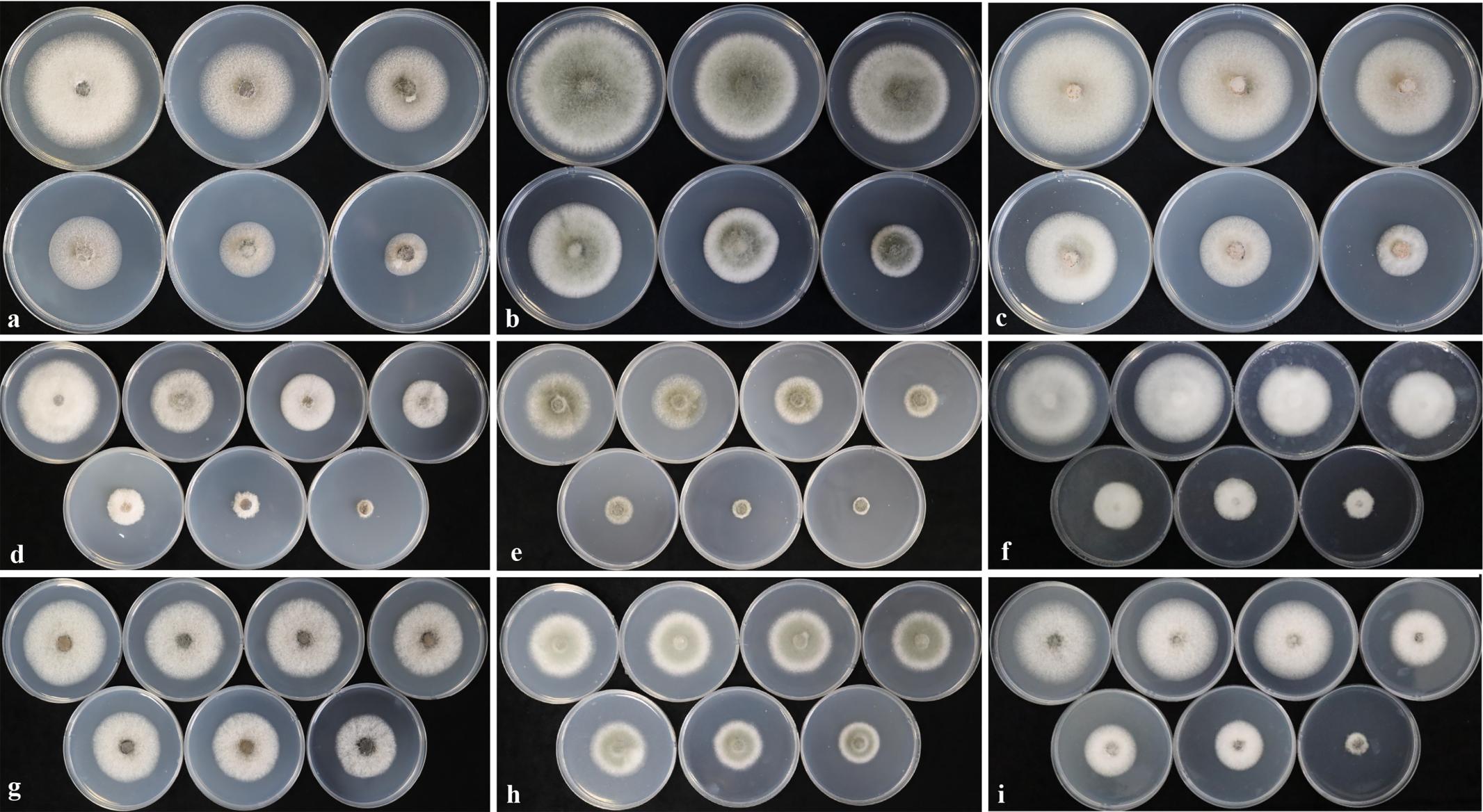
**

**Supplementary Figure S3** The sensitivity of three dominant *Colletotrichum* species to the tested fungicides. a-c. Prochloraz; d-f. Difenoconazole; g-i. Tebuconazole; a, d, g. *C. gloeosporioides*; b, e, h. *C. fructicola*; c, f, i. *C. aenigma*
